# Supplementary material for: Artificial intelligence model for analyzing colonic endoscopy images to detect changes associated with irritable bowel syndrome
Source: PLOS Digit Health. 2023 Feb 17;2(2):e0000058. doi: 10.1371/journal.pdig.0000058 (PMC9937744; doi:10.1371/journal.pdig.0000058)
Supplement: Attachment — Submitted filename: Response to Reviewers.docx [file pdig.0000058.s001.docx]

**Benjamin P. Geisler, M.D., M.P.H., F.A.C.P., M.R.C.P. (London), F.H.M.
Academic Editor
*PLoS Digital Health***

Dear Dr. Geisler:

On behalf of my co-authors, I thank you and the reviewers for considering our submission, *Artificial Intelligence Model for Analyzing Colonic Endoscopy Images to Detect Changes Associated with Irritable Bowel Syndrome*, for publication in *PLoS Digital Health*. We believe that the points raised in the careful and comprehensive evaluation of our manuscript have helped substantially strengthen our study. Following please find a point-by-point response to the concerns raised by you and the reviewers. We are hopeful that the revised manuscript is now suitable for publication.

Please let me know if there is any additional information that I can provide.

Warmest regards,

Dr. Hiroshi Mihara

University of Toyama

**Journal Requirements:

1. In the online submission form, you indicated that “Data cannot be shared for confidentiality reasons. Queries about the data should be directed to the corresponding author.”. All PLOS journals now require all data underlying the findings described in their manuscript to be freely available to other researchers, either 1. In a public repository, 2. Within the manuscript itself, or 3. Uploaded as supplementary information.
This policy applies to all data except where public deposition would breach compliance with the protocol approved by your research ethics board. If your data cannot be made publicly available for ethical or legal reasons (e.g., public availability would compromise patient privacy), please explain your reasons by return email and your exemption request will be escalated to the editor for approval. Your exemption request will be handled independently and will not hold up the peer review process, but will need to be resolved should your manuscript be accepted for publication. One of the Editorial team will then be in touch if there are any issues.**

At the time of submission, we provided two datasets from a cloud service (Google Drive). We now also provide two other datasets and include links to them in the revised manuscript. In addition, we updated the revised version with an explanation regarding the availability of data. Several images were excluded due to ethical considerations, although they were used in model building.
 **2. Please provide separate figure files in .tif or .eps format and remove any figures embedded in your manuscript file. Please also ensure that all files are under our size limit of 10MB.

For more information about how to convert your figure files please see our guidelines:** [**https://journals.plos.org/digitalhealth/s/figures**](https://journals.plos.org/digitalhealth/s/figures)

We now provide each figure as a .tif file that does not exceed the 10 MB limit. The revised version now includes figure titles.

**3. Please ensure that you refer to Table 3 in your text as, if accepted, production will need this reference to link the reader to the table.**

We apologize for this oversight. In the original version, Table 2 was listed twice. In the revised version, the second table is now labeled as Table 3.
 **4. All figures and supporting information files will be published under the Creative Commons Attribution License (**[**creativecommons.org/licenses/by/4.0/**](http://creativecommons.org/licenses/by/4.0/)**). Authors retain ownership of the copyright for their article and are responsible for third-party content used in the article.

Figures 2, 3, and 6: Please confirm (a) that you are the photographer; or (b) provide written permission from the photographer to publish the photo(s) under our CC-BY 4.0 license.

Please upload any written confirmation as an 'Other' file type. It must clarify that the copyright holder understands and agrees to the terms of the CC BY 4.0 license; general permission forms that do not specify permission to publish under the CC BY 4.0 will not be accepted. Note that uploading an email confirmation is acceptable.**

Figures 2, 3, and 6 were created by the first author, who has agreed to release them under the CC-BY 4.0 license. **Reviewers' comments:**

**Reviewer #1:**

**The authors’ purpose was to determine whether image AI models can differentiate between different types of irritable bowel syndrome (IBS) and healthy colonoscopic images using Google cloud AutoML Vision.
I have some comments to make on their research.**

We thank Reviewer 1 for their thorough evaluation of our manuscript. We have responded to each point raised as described below.

**1.      Page 6, line 90-92: I think that that the text and references do not match. The authors should check this.**

**2.      Page 7, line 98: The content of the reference and the text appear not to match.**

**3.      Page 7, line 98-100: The content of the references clearly does not match the text.**

For Points 1-3, we thank you for noting this issue. During editing, the order of some references was changed. We have updated the citations in the revised version so that all the references now correspond to the text.

**4.      Page 8, line120-122: The authors mention some groups of patients. However, they do not give any background information for them. The authors should state such background factors as gender, age, history of illness, and treatment history. In addition, I think that the total numbers of patients are not sufficient to make an AI model.**

For the colonoscopy images that were used, some patient characteristics such as gender and age were not known. As such, these factors were not considered during construction of the AI model. Since these characteristics may be confounding factors for AI construction, we updated the text to reference this as a potential limitation of the study that should be addressed in future research. In addition, we note that the Google AutoML service explicitly states that an AI model can be constructed using 100 images for each group.

**Reviewer #2: In this retrospective study from Japan, Mihara et al used an artificial neural network (ANN) AI classifiers to analyze and model endoscopic images from colonoscopies to differentiate between patients with various irritable bowel syndrome/IBS subtypes (IBS, IBS-D, IBS-C) versus healthy controls with relatively good performance with an area under the curve (AUC) ranging from 0.85-0.93. Objective markers of diagnosing IBS are much needed. This is an interesting study and addresses an important topic. I have the following critiques and recommendations:**

We thank Reviewer 2 for their thorough evaluation of our manuscript. We have responded to each point raised as described below.  **1. Abstract, Background/Aims: "IBS is not an organic disease, and the patients typically show no abnormalities in lower gastrointestinal endoscopy." The authors are correct that IBS patients usually have normal colonoscopies (endoscopic and histologic). Since most colonoscopies are normal in patients with IBS, the authors should clarify why they chose to review colonoscopy images by AI modeling.**

As mentioned in the abstract, IBS does not show abnormalities on lower gastrointestinal endoscopy, but biofilm formation and histological microinflammation are known to occur. Thus, we tentatively assumed that AI machine learning could be used to capture minute changes on endoscopic images that would be judged as normal by the human eye. We have revised the text to better convey this concept.

**2. Abstract, Background/Aims:  "Recently, biofilm formation has been visualized by endoscopy, and the ability of endoscopy to detect microscopic changes due to dysbiosis and microinflammation has been reported."  If this is the case, why did the authors only look at patients with IBS without biofilms? Why mention biofilms if their study doesn't even measure this?**

Since it is currently not possible for the human eye to determine whether an endoscopic image was taken from a patient with IBS, even if microinflammation is present histologically, we have revised the abstract and introductory description so that biofilm is mentioned only as a possible microscopic feature of IBS. The Methods are updated to indicate that biofilm was not detected in the samples used in this study. **3. Methods: "These names included "Irritable bowel syndrome (group I)." The authors should clarify what type of IBS patients were included in Group 1 and how they differed from the other groups with constipation-predominant (IBS-C) and diarrhea-predominant (IBS-D).**

Our first question was whether AI could be used to distinguish Group I from Group N. Irritable bowel syndrome includes IBS-C, IBS-D, and IBS-MIX, but we did not examine the proportions of these types within Group I. In addition, patients prescribed ramosetron or linacrotide would be classified as Group D or Group C. As such, Group I comprised patients who were not prescribed either of these drugs.

**4. Methods: Among patients with IBS-D, were other etiologies of chronic diarrhea ruled out (e.g. inflammatory bowel disease, microscopic colitis, small intestinal bacterial overgrowth)?**

Patients with colorectal cancer and/or other diseases such as inflammatory bowel disease and eosinophilic gastroenteritis were excluded from the study. We have revised the text to note this exclusion.  **5. Methods: The authors should clarify the rationale for colonoscopy in the included patients? Was this screening colonoscopy for colon cancer? Was colonoscopy done as part of workup of altered bowel habits (e.g. diarrhea)?**

Screening of symptomatic patients was done as part of a workup for changes in bowel habits (e.g., diarrhea), whereas asymptomatic patients had undergone routine colorectal cancer screening. **6. Results: The authors should include a Table 1 with baseline clinical characteristics of included patients and healthy controls include patient age, sex, medications, etc.**

To our knowledge, patient characteristics such as gender or age have not been reported to represent a significant factor in findings on colonoscopic images. As such, patient characteristics were not obtained for this study. On the other hand, when building a predictive AI model of whether a laxative response can be predicted based on background factors such as gender and age, more than 1,000 data sets of teaching data for each factor, together with endoscopic images, are needed. We plan to compile such sets for future studies. In this study, we have determined that obtaining data sets containing patient characteristics such as gender was not needed to address the question of whether AI can identify differences between IBS and healthy subjects.

**7. Methods: The authors should expand the details of the AI classifier they used for the study. Was ANN used versus other AI classifiers? What are the benefits of this strategy when analyzing endoscopic images?**

Several deep CNN architectures exist. The revised version now references other classifiers: 1)The predecessor model to GCP AutoML, GoogLeNet, which in 2014 won the ImageNet Large Scale Visual Recognition Competition (ILSVRC), an international image AI competition, by achieving high accuracy with low computational cost; 2) GCP AutoML, which is based on the theoretical background of the Neural Architecture Search and optimizes the neural network architecture itself. GCP AutoML represents a service that enables generation of high-quality image classification models even by individuals who have no extensive expertise in machine learning; and 3) Resnet, which enables construction of deep neural networks based on radiological images having up to 1,000 or more layers, and achieved better results than GoogLeNet. Furthermore, we note the need for future study to determine whether Microsoft Azure, which is based on Resnet for building code-free AI models for endoscopic images, performs better than GCP AutoML Vision. We updated the reference list to include the following citations:

1. Khan, A., Sohail, A., Zahoora, U. *et al.* A survey of the recent architectures of deep convolutional neural networks. *Artif Intell Rev* **53,** 5455–5516 (2020). <https://doi.org/10.1007/s10462-020-09825-6>
2. Ioffe S, Szegedy C. Batch Normalization: Accelerating Deep Network Training by Reducing Internal Covariate Shift. In: Francis B, David B, editors. Proceedings of the 32nd International Conference on Machine Learning; Proceedings of Machine Learning Research: PMLR; 2015. p. 448--56.
3. Ananda, A., Ngan, K.H., Karaba ˘g, C., Ter-Sarkisov, A., Alonso, E., Reyes-Aldasoro, C.C.

Classification and Visualisation of Normal and Abnormal Radiographs; A Comparison between Eleven Convolutional Neural Network Architectures. *Sensors*, **21**, 5381 (2021).

https://doi.org/10.3390/s21165381
 **8. Methods/Results: For the Figures, the author state "High-scoring images in patients with IBS (score 0-1)." What do these scores refer to? How was this derived and what do they mean clinically?**

The probability of a label for each image is presented as a score between 0-1.
The significance of the difference in scores is unclear at this time, but we expect that this point will be clarified as the studies progress.

**9. Methods/Results: What endoscopic features (e.g. alterations in vascular patterns, mucosal folds, colonic mucosa patterns or color, presence or scarring or erythema) did the artificial neural network (ANN) AI classifier use to differentiate/model patients with IBS vs healthy controls?**

The endoscopic features the Artificial Neural Network (ANN) AI classifier uses to distinguish/model IBS patients vs. healthy controls are unclear and the current model is effectively a black box. However, an explainable AI model is becoming available (Quellec et al., see citation below that now appears as reference #16 in the revised text). By adding a function to display areas of importance for the score, we can now determine which endoscopic features are characteristic of IBS.

1) Quellec G, Al Hajj H, Lamard M, et al. ExplAIn: Explanatory artificial intelligence for diabetic retinopathy diagnosis. *Med. Image Anal.* **72**, 102118 (2021). <https://doi.org/10.1016/j.media.2021.102118>.

**10. Methods/Results: Older patients with chronic constipation often have diverticulosis across the colon. How did the AAN AI classifier account for diverticulosis in the predictive model? Did diverticulosis predict IBS-C? Similarly, patients who use laxatives sometimes have discoloration of the colon (melanosis coli). How did the AAN AI classifier model this finding?**

The groups were not defined according to laxative use, and the presence of diverticula or colonic melanosis was not taken into account during model construction. The extent to which these factors could affect the score values will become clearer when an explainable AI is constructed in the future.

**11. Methods/Results: Were biopsies obtained from the included patient and histologic assessment performed to rule out microscopic colitis? Sometimes during colonoscopy, colonic mucosa appears endoscopically normally, but has inflammation at the histologic level.**

Patients who were biopsied for diarrhea and other conditions and diagnosed with eosinophilic gastroenteritis or other conditions were excluded. Mild inflammatory cell infiltration can be seen in healthy subjects and those with IBS, and if they did not meet the diagnostic criteria for other diseases, they were included in the study population (irritable bowel syndrome, diarrhea-type irritable bowel syndrome, constipation-type irritable bowel syndrome, and healthy subjects).

**12. Methods/Results: Different segments of the colon (right colon, transverse, rectum, etc) have different endoscopic features. For all the included patients, the authors should clarify how many endoscopic images were obtained for each segment and clarify whether images from specific segments were more diagnostic/contributed more to AI-derived model to differentiate IBS patients from healthy controls.**

In the text we note that we used a total of 20 to 40 images, with about five for each segment of the cecum, ascending, transverse, descending, and sigmoid colons, and the rectum. Since the model was not constructed by segment, whether different segments have different contributions to the diagnosis is unclear. It is assumed that IBS scores that are appropriate for different segments can be calculated. The advantage of our model is that it returns an IBS score that is independent of information about the segment from which the image was taken. Preliminary results show the greatest difference for the sigmoid colon, but additional detailed studies are needed before conclusions about the significance of contributions from different regions can be made.

**13. Methods/Results: The analysis could be more robust and not only differentiate/classify IBS vs non-IBS patients, but also should correlate the endoscopic images with validated IBS severity scores (e.g. Birmingham IBS Symptom Questionnaire , IBS Symptom Severity Scale, etc).**

Thank you for this very important suggestion. The purpose of this study was to determine whether a classification model based on image data alone could detect IBS. A prediction model including numerical data such as severity score would require at least 1000 datasets, and we plan to build a prediction model that includes both table and image data after collecting additional data.
 **14. Methods/Discussion: The authors should state that the small sample size and lack of validation of the ANN AI model in an independent cohort of patients are additional limitations of the study.**

Although the minimum sample size for GCP AutoML Vision is 100 images, ideally more than 1000 images are required. The fact that the training, validation, and test images are from the same patient population, even though very different images were used, is noted as one of the limitations of this study. Validation of the method using an independent patient cohort is needed.

**15. Discussion: The authors state "Endoscopic images of IBS typically show no abnormalities, but we investigated whether AI could detect microinflammation that cannot be easily detected by human observers."  How was microinflammation defined and assessed from colonoscopy endosocopic images in the included cohort of patients?**

We updated the text to note that the histopathology had not been reviewed and thus is referenced only in general terms:

Endoscopic images of patients with IBS typically show no abnormalities. In this study, we investigated whether AI can detect minute endoscopic changes associated with microinflammation and other microenvironmental changes that cannot be easily detected by human observers. **Reviewer #3: The authors applied the Google cloud platform AutoML Vision to build a model to differentiate between patients with IBS and asymptomatic healthy controls based on colonoscopy images. The validation cohort was a subset of the training set and the model could differentiate asymptomatic controls and IBS patients with a sensitivity of 30 % and specificity of 97 %.**

**Before publication some major points need to be clarified.**

We thank Reviewer 3 for their thorough evaluation of our manuscript. We have responded to each point raised as described below.

**- The “group I” comprised of “IBS” patients while “group C” and “D” comprised of IBS-C and IBS-D patients. While IBS-D and IBS-C are clear subgroups, it is unclear, what defines group “I”. Also the diagnostic algorithm for IBS diagnosis is not well defined. Rome criteria should be applied. For the sake of clinical relevance we recommend combining IBS-M and IBS-D and excluding IBS-C from such analysis. Also a larger dataset (>100 patients and controls) would reduce overfitting.**

In the future, approximation for each group may also be useful in elucidating the pathophysiology by examining the accuracy of the AI that distinguishes IBS-M, IBS-D, IBS-C, and healthy subjects using the ROME criteria, or by comparing the accuracy of AI among mixed groups of patients.

**- The authors show colonoscopy pictures of high scoring IBS and control patients (Figure 2 and 3). There is no evident visual difference between those pictures, but authors suggest that a machine learning model might pick up differences in i.e. vasculation pattern. In fact endoscopically visible biofilms as mentioned in their introduction are obviously not present in the representative images. Were any biofilms detectable in this study cohort ? Were images with incomplete bowel cleansing removed from the data set ? Then they likely have remove obvious biofilm patients from their data set.**

The Methods were updated to indicate that no biofilm was detected and that images with scores of 2 (minor amount of residual staining, small fragments of stool and/or opaque liquid, with good visualization of mucosa in the colon segment) or 3 (entire mucosa of the colon segment was well-visualized with no residual staining, small fragments of stool and/or opaque liquid) on the Boston Bowel Preparation Scale (BBPS)1) were employed.

1) Lai E.J. et al. The Boston Bowel Preparation Scale: A valid and reliable instrument for colonoscopy-oriented research. *Gastrointest Endosc*., **69**, 6020-625. (2009). doi:10.1016/j.gie.2008.05.057.

**-Machine learning is highly sensitive to bias. Authors need to improve on cohort characterization; were all patients scoped with the same model of endoscope. Where patients from each cohort scoped in the same hospital/ by the same endoscopist?**

We updated the Methods to indicate that the images were taken by more than 10 endoscopists and specialists working at a single institution and using Olympus CF-HQ290Z or PCF-H290Z colonoscopes.

**Minor points:
-Abstract, Background and Aims: authors start with “IBS is not an organic disease...” but continue to elaborate on biofilm formation, dysbiosis and microinflammation. This mismatch needs to be elaborated.**

Corrected to: "IBS is not considered to be an organic disease…"

**Reviewer #4: The authors made a image recognition model using Google AutoML vision. They successfully distinguished healthy vs IB”D”(probably “S”) samples.

The approach has merits and significant advantage in understanding the pathophysiology of IB”D”(probably “S”).

However, the authors are using Google AutoML tool as a black box. The training and validation statistics are only limited the to final ROC or contingency tables.

I think the authors should reveal how training improved the models in different iterations. They should clearly separate traning and validation datasets with number of samples.**

We thank Reviewer 4 for his/her thorough evaluation of our manuscript. We have responded to each point raised as described below.  **The tables with AUC should also reveal number of samples used.

The authors didn't reveal how the images were scores and how were they ranked.**

The accuracy of the model was improved by building the model multiple times after excluding images of the terminal ileum, dye images, magnified observations, rectal inversion images, proximity images, and blurred images. We reference in part the number of data samples for training, validation, and testing, and added the number of data samples used for all models.

The number of samples for each model is now clearly stated.

Reviewer 2 also raised this point and in the revised version we state that the scores are automatically calculated with the likelihood being between 0 and 1.
