# Supplementary material for: Artificial intelligence model for analyzing colonic endoscopy images to detect changes associated with irritable bowel syndrome
Source: PLOS Digit Health. 2023 Feb 17;2(2):e0000058. doi: 10.1371/journal.pdig.0000058 (PMC9937744; doi:10.1371/journal.pdig.0000058)
Supplement: Attachment — Submitted filename: Response to Reviewers.docx [file pdig.0000058.s002.docx]

**Benjamin P. Geisler, M.D., M.P.H., F.A.C.P., M.R.C.P. (London), F.H.M.
Academic Editor
*PLoS Digital Health***

Dear Dr. Geisler:

On behalf of my co-authors, I thank you and the reviewers for considering our 1^st^ revision, *Artificial Intelligence Model for Analyzing Colonic Endoscopy Images to Detect Changes Associated with Irritable Bowel Syndrome*, for publication in *PLoS Digital Health*. We believe that the points raised in the careful and comprehensive evaluation of our manuscript have helped substantially strengthen our study. Following please find a point-by-point response to the concerns raised by you and the reviewers. We are hopeful that the 2^nd^ revised manuscript is now suitable for publication.

Please let me know if there is any additional information that I can provide.

Warmest regards,

Dr. Hiroshi Mihara

University of Toyama

**Reviewers' comments:**

**Reviewer #1: This revised paper is well written and acceptable to be published in PLOS Digithal Health.
Reviewer #2: The authors have addressed my critiques to the best of their available datasets.**
Thank you very much for your confirmation.

**Reviewer #3: The authors did not perform the requested analysis of all IBS patients combined against controls. Splitting IBS patients into small ill-defined groups and comparing against a bigger cohort increases the possibility of overfitting. In terms of study design neither image location, endoscopic equipment nor disease classification was standardized. Furthermore, the authors could not specify relevant changes such as endoscopically visible biofilms in their images. An independent control cohort, which underlines clinical applicability of the model is lacking. It is thus more likely that the model is able to detect a signal for individual patients instead of underlying features of IBS.**

Thank you for pointing this out. We have constructed the model in the same way between the combined images of the IBS, IBS-C, and IBS-D groups in this study and the normal group (Group N), and have added the results in the revised manuscript as followed.

In result section:

Group N vs. Group I + Group C +Group D

Finally, Groups I, C, and D as a whole were merged to see if they could be distinguished from Group N using the same images. The average precision (positive predictive value), precision, and recall of the algorithm were 81.2%, 72.6%, and 72.6%, respectively.

In discussion section:

The model for Group N vs. Group I + Group C + Group D is slightly less accurate than Group N vs. Group I, suggesting that Groups I, C, and D may be different populations in terms of images..

The ultimate goal of this study is to construct a model that will be useful for treatment strategies in clinical settings with different imaging methods, endoscopic equipment, and endoscopists. In addition, although the diagnosis should be made with ROME IV, the transition to ROME V is being considered in the future, and in this study, the clinical classification of IBS-C group with high prevalence of linaclotide prescription, IBS-D with high prevalence of lamosetron prescription, and the other IBS were selected. In the future, it will be necessary to construct image AI according to each ROME criteria and to compare them.

**Reviewer #4: The authors have not addressed my question of providing iterative improvements of the ML models during training phase. This data is crucial because it will show if the models are converging and achieving optimal path or no change in improvements at all. This is usually done by showing a plot of Loss vs Epochs or Accuracy vs Epochs.**

Thank you for pointing this out. We apologize for the inconvenience of listing this in a slightly distant part of the text rather than immediately below the question, and for not putting it in red in the manuscript.

In our previous response, we stated the following:

**Reviewer #4: I think the authors should reveal how training improved the models in different iterations. They should clearly separate traning and validation datasets with number of samples.**

Our previous answer:

The accuracy of the model was improved by building the model multiple times after excluding images of the terminal ileum, dye images, magnified images, rectal inversion images, proximity images, and blurred images.

The following description has been added to the revised manuscript:

The accuracy of the model was improved by building the model multiple times after excluding normal light images of the terminal ileum, rectal inversion and anus, narrow band or dye-spread images.

In addition, the following was added to the limitation this time:

And GCP AutoML Vision does not allow iterative refinements during the training phase in the form of loss vs. epoch or accuracy vs. epoch plots, making it difficult to confirm that the model is converging on the optimal path.
